# Exposure assessment and cancer risk characterization of aflatoxin M_1_ (AFM_1_) through ingestion of raw cow milk in southern Ghana

**DOI:** 10.1016/j.toxrep.2022.05.015

**Published:** 2022-05-20

**Authors:** Nii Korley Kortei, Theophilus Annan, Vincent Kyei-Baffour, Edward Ken Essuman, Adjoa Agyemang Boakye, Clement Okraku Tettey, Nathaniel Owusu Boadi

**Affiliations:** a Department of Nutrition and Dietetics, School of Allied Health Sciences, University of Health and Allied Sciences, PMB 31, Ho, Ghana; b Food Microbiology Division, Council for Scientific and Industrial Research, Food Research Institute, P.O. Box M20, Accra, Ghana; c Food Chemistry and Nutrition Research Division, Council for Scientific and Industrial Research, Food Research Institute, P.O. Box M20, Accra, Ghana; d Department of Biomedical Sciences, School of Basic and Biomedical Sciences, University of Health and Allied Sciences, PMB 31, Ho, Ghana; e Department of Chemistry, Kwame Nkrumah University of Science and Technology, Kumasi, Ghana

**Keywords:** Aflatoxin M1 (AFM_1_), Toxigenic fungi, Raw milk, Mycotoxins, Ghana, HPLC-FLD

## Abstract

Milk and dairy products are the most important nutritional foods among all age groups. Aflatoxin M1 (AFM_1_) contaminates milk and makes its consumption potentially dangerous. Infants are mostly at risk because they are typically fed as many as six and more times per day, which is indeed a disquieting concern. This study aimed at evaluating AFM_1_ levels especially above international (European Food Safety Authority, EFSA) (0.05 µg/kg) and local (Ghana Standards Authority, GSA) (0.5 µg/kg) standards and cancer risks associated with the ingestion of raw cow milk (n = 120) sampled from Southern Ghana (Greater Accra, Volta, Western and Eastern Regions). AFM_1_ were measured with High-Performance Liquid Chromatography with a Fluorescence Detector (HPLC-FLD). Risk assessments were also conducted using models prescribed by the Joint FAO/WHO Expert Committee on Additives (JECFA). Out of the 120 samples analyzed for AFM_1_, 67 (55.8%) tested positive, 63 (52.5%) exceeded the limits of EFSA and were between the range 0.06 ± 0.001–3.52 ± 0.5 µg/kg whereas 50(41.7%) within the range of 0.50 ± 0.03–3.52.01 ± 0.5 µg/kg exceeded GSA limits. Risk assessments of AFM_1_ for infants, toddlers, children, adolescents, and adults ranged between 0.06 and 2.03 ng/kg bw/day, 197.04–6666.67, 0–0.0323 ng aflatoxins/kg bw/day and 1.94 × 10^-3^- 0.07 cases/100,000 person/yr respectively for Estimated Daily Intake (EDI), Margin of Exposure (MOE), Average Potency, and Cancer Risks. It was concluded that the consumption of raw milk posed adverse health effects on all age categories studied for the regions investigated. The use of raw cow milk may cause some problems and endanger the health of people of different age groups due to noncompliance with prescribed regulatory limits.

## Highlights

• Overall 55.8% were positive for AFM1 in 120 raw cow milk samples analyzed in Southern Ghana.
• 41.7% and 52.5% were recorded as contaminated raw cow milk samples exceeding permissible limits.
• Risk assessment on raw cow milk samples revealed a public health concern.
• Exposure to AFM1 and vulnerability principally result from the consumption of fungi infected.

## Graphical Abstract

**Figure:**
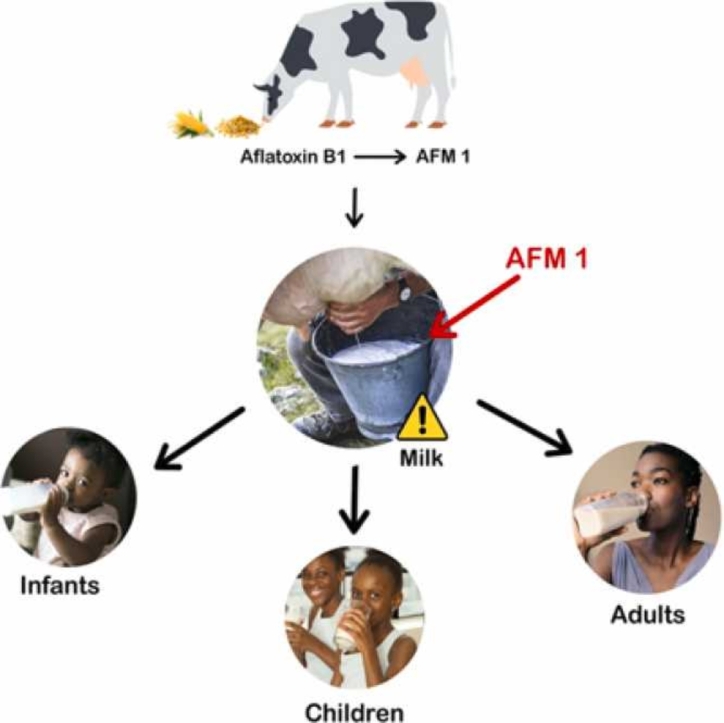

## 1 Introduction

Milk is a superb source of nutrients, being an aqueous colloidal suspension containing countless macro-and micronutrients that are essential for the growth, supply of energy, reproduction, maintenance and repair, appetite satisfaction and maintenance of human health [91], [74]. In spite of the health benefits of milk, mycotoxins are one probable group of natural food contaminants which contaminate milk and render it unsafe for consumption when ingested, which may cause ailments and pose adverse health effects.

Mycotoxins are natural toxins from fungi which contaminate a wide range of foodstuffs and render them potentially dangerous.

Mycotoxins represent one of the main global foodborne risks for human health and are considered an important issue in the situation of food safety, due to their acute and chronic toxic effects on animals and humans [11], [7].

Aflatoxins are mycotoxins produced by strains of *Aspergillus flavus* and *A. parasiticus*. Rao et al. [107] highlighted that they are comparatively the most known mycotoxins owing to their ubiquitous nature and persistence of *Aspergillus sp*. in the environment. Just about 4.5 billion people globally are at risk of disproportionate exposure to aflatoxins, which account for 4.6–28.2% of all cases of hepatocellular carcinoma [3]. Aflatoxins occur as five different types; aflatoxins B_1_, B_2_, G_1_, G_2_, and M_1_ produced primarily in cow milk by cows eating contaminated silage.

AFM_1_ contamination of milk and milk products at the global level is well established and has been reported in many countries [54]. AFM_1_ is a hydroxylated metabolite of AFB_1_ [15], that is, excreted in milk in the mammary glands of both humans and lactating animals [31], [32]. AFB_1_ is reported to have a significant association with AFM_1_ and so it is possible to predict the outcome of AFM_1_ with knowledge of AFB_1_ [68]. Various studies in recent decades have been committed to reducing the bioavailability of AFB_1_ thereby reducing the levels of AFM_1_ in lactating dairy cows [68]. Approximately 0.3–6.2% of AFB_1_ is converted into metabolized AFM_1_ and excreted in milk, depending on factors such as the genetics of the animals, seasonal variation, the milking process and environmental conditions [106]. In spite of AFM_1_’s comparatively lower of about 10 times its carcinogenic potential as AFB_1_, it still can be a probable health hazard to humans, especially children, considering their high milk consumption, lower body weight, high metabolic rate, and incomplete development of excretory organs [66].

Globally, studies have also shown that the presence of AFM_1_ in milk and dairy products is an important health issue because in many countries, every age group regularly consumes these products in their daily diet [32], especially in developing countries [86]. Children and especially infants are among the human population, who require milk for proper development during growth and often consume milk in greater quantities [85]. Besides milk consumption, their propensity of exposure to aflatoxins is further increased through the consumption of complementary feeds prepared from cereals and legumes [5], [56], which are foodstuffs prone to contamination by aflatoxigenic fungi (*Aspergillus* species). These complementary foods are formulated with milk again or consumed with milk.

AFM_1_ are dispersed universally but are commonly found in tropical or subtropical climates where there is an extreme range of temperature, rainfall, and humidity [30]. AFM_1_ contamination can also occur during the processing and storage of milk and its products. However, it has the ability to withstand numerous ordinary methods of food treatment such as cooking, sterilization, and freezing, hence its appearance in milk and dairy products [83]. Novel methods for minimizing the occurrence of AFM_1_ in milk by engaging yeast and lactic acid bacteria (LAB) microbial extracts as well as lactic acid fermentation to ferment a variety of traditional foods have shown aptitude for aflatoxin degradation, nevertheless are yet to be completely discovered [27], [53], [71].

Additionally, prolonged consumption of aflatoxins is well known to cause impaired immune function, teratogenic mutagenic disability [97], [35]. Aflatoxins have been reported to be associated with marasmus and kwashiorkor in children [99] and present some neurotoxic and neuro-immunotoxic effects of aflatoxin B_1_ as reported by Richard et al. [88]. Most importantly, aflatoxins have been reported to work concomitantly to worsen the risk of hepatocellular carcinoma (HCC), which is acknowledged to be the fifth most frequently occurring cancer in the world [44], [64]. A previous study by Liu and Wu [65] estimated that about 5–28% of global HCC cases are attributable to aflatoxin exposure, which occurs mainly through diet.

In view of these predicaments posed by mycotoxins (aflatoxins), limits are usually set by countries; eg. Europe (EFSA= 0.05 µg/kg) and other African countries such as Ghana (GSA= 0.5 µg/kg) to regulate the permissible levels to safeguard consumers from HCC attributable to AFM_1_ and some other negative health effects. In addition to setting regulatory limits for mycotoxins, it is also imperative to conduct health risk assessments in the population due to dietary exposure. A low-dose extrapolation approach introduced by the Joint FAO/WHO Expert Committee on Food Additives (JECFA) in 1997 and the margin of exposure (MOE) method proposed at the 64th JECFA meeting in 2005 [16] were both recommended and have been widely used worldwide [100], [48] to assess the risk of dietary exposure to mycotoxins.

In Ghana, insufficient research has been done on the prevalence of AFM_1_ in milk and milk products. To the best of our knowledge, this work is the only one with the objective of determining the levels, dietary intake assessment, and cancer risk involved with the ingestion of AFM_1_ through raw cow milk in southern Ghana. The outcome of this research will be expedient in informing policy makers on where to focus regarding food safety, particularly fungal intoxication of foods. Again, in adopting international legislations on food quality parameters. Lastly, contamination of raw cow milk with AFM_1_ should be considered a high priority for Ghana’s mycotoxin risk management actions.

## 2 Materials and methods

### 2.1 Chemicals and standards

The analytical standard of AFM_1_ was supplied by Sigma-Aldrich (St. Louis, MO, USA). All solvents used for the preparation of the mobile phase were HPLC grade and obtained from Merck (Darmstadt, Germany). All homogenized mixtures and eluates were filtered through Whatman no. 4 and 0.45 mm membrane filters, respectively (Whatman plc, Maidstone, UK). De-ionized water was obtained with a Millipore Elix Essential purification system (Bedford, MA, USA). EASI-extracted AFM_1_ immunoaffinity columns (stored at 4 °C until use) were supplied by R-Biopharm, Rhone limited and used for SPE and cleanup.

### 2.2 Preparation of standard solutions

A mother stock solution (0.1 μg/mL) was prepared from a standard solution of AFM_1_ (0.993 μg/mL in acetonitrile) and stored with care in a freezer. A working stock solution of 0.01 μg/mL was diluted step by step with the combined solution (acetonitrile/water, 75/25, v/v) to prepare a sequence of working solutions which were stored in vials below 4 ºC for the calibration curve. Calibration solutions of 0.02 μg/kg, 0.04 μg/kg, 0.06 μg/kg, 0.08 μg/kg, and 0.10 μg/kg were used. Samples with AFM_1_ amount above the calibration range were diluted and dilution factors applied for quantification as outlined by EN ISO 14501:2007 [101].

## 3 Materials

A total of one hundred and twenty (n = 120) raw cow milk samples were used in this study. Thirty (30) raw cow milk samples were obtained from local markets in each region; Greater Accra, Volta Region, Western and Eastern Regions of Ghana (Table 1) between the period of March and July 2021. Milk samples were stored in coolers with ice packs and transported to the laboratory and analyzed for AFM_1_.

**Table 1:** Geographical location and some attributes of the origins of raw cow milk samples.

Region	No. of samples	Agro-ecological zones	Rainfall (mm)	Temperature (^o^C)	Coordinates
Greater Accra	30/120	Coastal Savannah	800–1000	26.6	5.8143°N, 0.0747° E
Volta	30/120	Coastal Savannah/Deciduous Forest	1000–1400	26.2	6.5781°N, 0.4502° W
Western	30/120	Evergreen	1800–2000	25.9	5.3902°N, 2.1450°W
Eastern	30/120	Deciduous Forest	1400–1900	25.9	6.2374°N, 0.4502°W

### 3.1 Preparation of samples

After warming at about 37 ºC in a water bath, the samples were centrifuged at 2000 g to separate the fat layers and then filtered. The prepared test portion of 50 mL was transferred into a syringe barrel attached to AFM_1_ immunoaffinity column and passed at a slow steady flow rate of 1–2 mL/min. The columns were then washed with 20 mL deionized water and air was passed through the columns to dryness. AFM_1_ was eluted with 4 mL pure acetonitrile by allowing it to be in contact with the column for not less than 60 s. The eluate was evaporated to dryness using a gentle streams of nitrogen. The residue was dissolved in 500 µl of mobile phase and filtered using a membrane filter before injected into HPLC for quantification (EN ISO 14501:2007) [101].

## 4 Instrumentation

Agilent high performance liquid chromatography system (HPLC 1260 infinity series) with a quaternary pump and fluorescence detection was used for AFM_1_ quantification analysis and was carried out as per the method given by EN ISO 14501:2007 [101]. Data acquisition and quantification was done using Chem station (Open Lab edition). The Agilent HPLC equipped with a fluorescence detector was set at an excitation wavelength of 360 nm and an emission wavelength of 440 nm and the column compartment (HPLC Column: TC-C18 (2), 170, 5 µm, 4,6 × 250 mm; thus, pore size of 170, particle size of 5.0 µ, inner diameter of 4.6 mm, length of 250 mm and carbon load of 12%) temperature regulated at 35 °C. The mobile phase was a mixture of water and acetonitrile at ratios of 25:75 (v/v), respectively, and an isocratic delivery mode was employed at a flow rate of 0.8 mL/min with an injection volume of 10 µl.

## 5 Validation

HPLC-FLD method was validated according to the guidelines of European Commission Decision 657/2002/EC for confirmatory analysis methods and the tested parameters were linearity, limit of detection (LOD), limit of quantification (LOQ), accuracy, precision and selectivity. The linearity was assessed by constructing five-point solvent matched calibrations in triplicate for AFM_1_ standard solutions in the concentration range of 0.05–0.8 mg/L. Calibration curves were drawn by plotting the peak area against AFM_1_ concentration, and linearity was evaluated by linear regression analysis expressed as coefficient of determination (r^2^).

Precision of the method was estimated in terms of % RSD of three identical extractions of milk samples spiked with AFM_1_ at the same as well as at three different spiking levels. Method selectivity was evaluated by analyzing AFM_1_ known negative milk matrix and reagent blank to determine any interference from endogenous substances around the retention time of the target analyte.

## 6 Risk assessment of exposure to AFM_1_ via consumption of milk

### 6.1 Estimation of exposure

Estimated Daily Intake (EDI) was considered by using the mean quantities of aflatoxins derived from the milk samples, the quantity of sample consumed daily, and the average body weight. The EDI for mean aflatoxin was premeditated according to the following formula (1) and expressed in μg/kg of body weight/day (μg/kg b.w/day) [26], [92].

(1) EDI = *daily intake* (food) X mean level of AFM_1_ average bodyweight

Daily intake of milk in Ghana according to Omore et al. [76] is approximately 0.0137 kg/day (5.0 kg/year).

The different age categories according to EFSA (Panel on Dietetic Products and Allergies) (2009) and their corresponding estimated average weights in Ghana used in this study were done as follows; Infants- 2.9 (2.5–3.2) kg [4], [62], Toddler − 9.8 (7–12.6) kg [1], [39], Children − 26 (24–28) kg [19], [79], Adolescents- 46.25 (38.5–54) kg [10], Adults- 60.7 kg [108].

### 6.2 Margin of exposure characterization for aflatoxins

Genotoxic and carcinogenic compounds such as aflatoxins have their risk assessment fittingly computed based on the Margin of Exposure (MOEs) approach, which was estimated by dividing the Benchmark dose lower limit (BMDL) for aflatoxins 400 ng/kg bw/day by toxin exposure [9], [25], [29], [37] as expressed in Eq. (2).

(2) MOE= Benchmark dose lower limit EDI (Exposure)

The EFSA [24] identified the liver carcinogenicity of aflatoxins as the critical consequence of the risk assessment; therefore, the benchmark dose lower confidence limit for a benchmark response of 10% (BMDL_10_) regarding the frequency of hepatocellular carcinomas (HCCs) in male rats was considered. In the absence of a specific BMDL_10_ for AFM_1_, the BMDL_10_ for HCCs related to the ingestion of AFB_1_ (0.4 μg/kg or 400 ng/kg body weight per day) [25], [90] was used in the present study for the definition of MOE applying a potency factor. A public health alarm is raised in instances where MOEs are less than 100,000 [9], [37], [57].

### 6.3 Estimated liver cancer risk due to consumption of raw cow milk

The ingestion of aflatoxins can be linked to the onset of liver cancer [95]. Therefore, liver cancer risk estimation for Ghanaian adult consumers was calculated for aflatoxins [95]. This involved estimating the population cancer risk per 100,000, which is a product of the EDI value and the average hepatocellular carcinoma (HCC) potency figure from individual potencies of Hepatitis B surface antigen (HBsAg) (HBsAg-positive and HBsAg-negative groups).

The JECFA [38] estimated potency values for AFM_1_ which corresponded to 0.3 cancers /year/100,000 population ng/kg bw/day (uncertainty range: 0.05–0.5) in HBsAg-positive individuals and 0.01 cancer/year/100, 000 population/ ng/kg bw/day (uncertainty range: 0.002–0.03) in HBsAg-negative individuals [95] were adopted for this calculation. Furthermore, the average HBsAg+ prevalence rate of 7.74% (adult-8.36%, 14.3%-adolescents, 0.55%-children) in Ghana [2] was adopted and 92.26% (100–7.74%) was extrapolated for HBsAg-negative groups. Hence, the average potency for cancer in Ghana was estimated as follows according to Eq. (7) as prescribed by Shephard [95] and Adetunji et al. [9]:

(3) Average potency = [0.03 x HBsAg –negative individuals in Ghana] + [0.01 x HBsAg- positive individuals/prevalence rate in Ghana]

(0.3 × 0.077) + (0.01 × 0.9226).

= 0.0323.

Thus, the Cancer risk (cancers per year per 100,000 population per ng aflatoxin/kg bw/day) was estimated using the following formula in Eq. (8) [57], [9]:

(4) Cancer Risk = Exposure (EDI) × Average potency

## 7 Statistical analysis

The aflatoxin concentrations were calculated using regression analysis from the curves generated from the standards of AFM_1_ with Excel for Microsoft Windows (version 10). One sample *t*-test was used to compare the means obtained at a 95% confidence interval and 5% level of significance. The statistical results were summarized as median, standard deviation, variance, skewness, standard error of skewness, kurtosis and standard error of kurtosis and mean values (range from the 25th percentile to the 75th percentile). SPSS 22 (Chicago, USA) was used in the analysis of data. Deterministic risk assessment models were used; dietary exposure (Estimated Dietary Intake), MOE values, Average potency, and cancer risk.

## 8 Results

### 8.1 Occurrence of aflatoxins

Most of the food samples tested produced good linearity or coefficients of correlations (R^2^ > 0.990) within the tested range. The mean recovery percentage of AFM_1_ in spiked milk samples were found between 80.5% and 84.07% with % RSD from 3.19 to 5.42. Since the recoveries and % RSD were within the EC regulation.

The number of raw cow milk samples contaminated with AFM_1_ is presented in Table 2, Table 4. The level of occurrence of the AFM_1_ ranged between 0 and 2.61 µg/kg, 0–3.04 µg/kg, 0–3.52 µg/kg, and 0–2.11 µg/kg respectively for Greater Accra, Volta, Western and Eastern Regions. Greater Accra Region (GTA) recorded 0.26, 0.010, and 0.371 µg/kg for mean, median, and variance, respectively, while the skewness and kurtosis were 2.81 and 7.77 respectively and showed that the data set of AFM_1_ obtained in this town was asymmetrical and heavy-tailed (Table 2). The lower and upper limits were 0.031 and 0.486, respectively, and showed statistical differences (*P* < 0.05) (Table 3). For Volta Region (VR), values of 0.54, 0.00, and 0.65 µg/kg were recorded from the summary statistics as mean, median, and variance, respectively, while 1.59 and 2.13 were recorded as skewness and kurtosis and implied moderate skewness and light-tailed. The upper and lower limits were 0.234 and 0.837. Values significantly differed (*P* < 0.05) (Table 2, Table 3). The mean, median, and variance recorded for Western Region (W*R*) were 0.86, 0.63, and 0.89 µg/kg, respectively. While the data set showed symmetrical and light-tailed as, the skewness and kurtosis were 0.92 and 0.33, respectively (Table 2). Values of 0.503 and 1.206 were recorded as upper and lower limits. There were significant differences (*P* < 0.05) observed (Table 3). For Eastern Region (ER), the recorded mean, median, and variance were 0.67, 0.67, and 0.40 µg/kg, respectively. The data set for ER was fairly symmetrical and light-tailed (0.49 and −0.67 for skewness and kurtosis, respectively. Upper and lower limits of 0.431 and 0.902 were, respectively, recorded. There were statistically significant (*P* < 0.05) differences (Table 2, Table 3).

**Table 2:** Summary of statistics of AFM_1_ concentration in raw cow milk obtained from four different regions of Ghana.

	Greater Accra Region	Volta Region	Western Region	Eastern Region
No. of samples	30	30	30	30
Mean	0.26	0.54	0.86	0.67
Std. Error of Mean	0.111	0.147	0.172	0.115
Median	0.010	0.000	0.625	0.665
Std. Deviation	0.608	0.807	0.942	0.631
Variance	0.371	0.652	0.887	0.398
Skewness	2.814	1.590	0.916	0.494
Std. Error of Skewness	0.427	0.427	0.427	0.427
Kurtosis	7.769	2.133	0.334	-0.670
Std. Error of Kurtosis	0.833	0.833	0.833	0.833
Range	2.61	3.04	3.52	2.11
Percentiles 25	0.000	0.000	0.000	0.000
50	0.010	0.000	0.625	0.665
75	0.130	0.968	1.705	1.100

**Table 3:** Statistics of the AFM_1_ concentrations using one sample *t*-test of raw cow milk samples from different regions.

					95% confidence interval of the difference	
	t	df	Sig. (2-tailed)	Mean Difference	Lower	Upper
GTA	2.327	29	0.027	0.259	0.031	0.486
ER	5.787	29	0.000	0.667	0.431	0.902
VR	3.635	29	0.001	0.536	0.234	0.837
W*R*	4.969	29	0.000	0.855	0.503	1.2064

The European Food Safety Authority (EFSA) and Ghana Standards Authority (GSA) regulatory limits for AFM_1_ (Table 4) were used in this study. Toxin quantity thresholds prescribed by the Ghana Standards Authority are a subset of the European Food Safety Authority (EFSA). Regarding the frequency and (percentage %) of positive (Yes) AFM_1_ contaminated milk samples above the various permissible limits, The Greater Accra Region (GTA) recorded 13(43.3%) and ranged between 0.06 ± 0.001–2.61 ± 0.7 µg/kg and 4(13.3%) with 1.21 ± 0.15–2.61 ± 0.6 µg/kg tested positive for EFSA and GSA_,_ respectively. The overall AFM_1_ positive samples were 15 (50.0%). For Volta Region (VR), AFM_1_ values of 12(40.0%) which ranged between 0.55 ± 0.02–3.04 ± 0.7 and 12(40.0%) of 0.55 ± 0.02–3.04 ± 0.7 µg/kg. An overall positive AFM_1_ of 12(40.0%) was recorded. Western Region (W*R*) recorded total aflatoxin values of 18 (60%) within the range of 0.08 ± 0.001–3.52 ± 0.5 µg/kg and 17 (56.7%) within the range of 0.50 ± 0.03–3.52 ± 0.5 µg/kg were recorded. Values of 20 (66.7%) were recorded as overall positive AFM_1_ for the samples tested. In the Eastern Region (ER), AFM_1_ values of 20(66.7%) within a range of 0.09 ± 0.001–2.11 ± 0.4 µg/kg and 17(56.7%) within a range of 0.61 ± 0.02–2.11 ± 0.4 µg/kg were recorded. Values of 20(66.7%) were recorded as positive AFM_1_ among the samples. Out of the 120 samples analyzed for AFM_1_, 63% exceeded the limits of EFSA and were within the range of 0.06 ± 0.001–3.52 ± 0.5 µg/kg. While for GSA, 50 (41.7%) of samples exceeded and ranged between 0.50 ± 0.03–3.52 ± 0.5 µg/kg. Values of 67(55.8%) were recorded as the grand frequency and percentage of positive AFM_1_ among the samples.

**Table 4:** Proportions of AFM_1_ concentrations of raw cow milk samples that exceeded limits of the European Food Safety Authority (EFSA) and Ghana Standard Authority (GSA).

Region	No. of samples	Exceeding EFSA regulation		Exceeding GSA regulation		Overall positive with AFM_1_
		Yes (%)	Range	Yes (%)	Range	
Greater Accra	30	13 (43.3%)	0.06 ± 0.001–2.61 ± 0.7	4 (13.3%)	1.21 ± 0.15–2.61 ± 0.6	15 (50.0%)
Volta	30	12 (40%)	0.55 ± 0.02–3.04 ± 0.7	12 (40%)	0.55 ± 0.02–3.04 ± 0.7	12 (40.0%)
Western	30	18 (60%)	0.08 ± 0.001–3.52 ± 0.5	17 (56.7%)	0.50 ± 0.03–3.52 ± 0.5	20 (66.7%)
Eastern	30	20 (66.7%)	0.09 ± 0.001–2.11 ± 0.4	17 (56.7%)	0.61 ± 0.02–2.11 ± 0.4	20(66.7%)
*Total*	*120*	*63 (52.5%)*	*0.06 ± 0.001–3.52 ± 0.5*	*50 (41.7%)*	*0.50 ± 0.03–3.52 ± 0.5*	*67(55.8%)*

European Union Food Safety (EFSA) limit for AFM1 = 0.05 µg/kg

Ghana Standards Authority (GSA) limit for AFM_1_ = 0.5 µg/kg.

### 8.2 Risk assessment

The Estimated Daily Intakes (EDI) of total aflatoxins in the raw cow milk samples from Greater Accra (GTA) Region were 0.61, 0.36, 0.14, 0.08, and 0.06 ng/kg bw/day for infants, toddlers, children, adolescents, and adults respectively. The Margin of Exposure (MOE) values recorded were 655.74, 1111.11, 2857.14, 5000.00, and 6666.67, respectively. The average potency of the aflatoxins was 0.0323 aflatoxins ng/kg bw/day and produced cancer risks of 0.02, 0.01, 4.5 × 10^-3^, 2.58 × 10^-3^ and 1.94 × 10^-3^ cases/100,000 person/yr respectively (Table 5). Samples from Volta Region (VR) recorded EDI values of 1.28, 0.75, 0.29, 0.16, and 0.12 ng/kg bw/day for infants, toddlers, children, adolescents, and adults respectively. MOE values of 312.50, 533.33, 1379.31, 2500.00, and 3333.33. Average potency was same. Cancer risks of 0.04, 0.02, 9.37 × 10^-3^, 5.1 × 10^-3^, and 3.88 × 10^-3^ cases/100,000 person/yr respectively for these age categories were recorded (Table 5). In Western Region (W*R*), the EDI values recorded for infants, toddlers, children, adolescents, and adults were 2.03, 1.20, 0.45, 0.25, and 0.19 ng/kg bw/day respectively. MOE values recorded were 197.04, 333.33, 888.89, 1600.00, and 2105.26, respectively. The average potency was the same as other regions, while the cancer risks were 0.07, 0.04, 0.014, 8.08 × 10^-3^ and 6.14 × 10^-3^ cases/100,000 person/yr respectively (Table 5).

**Table 5:** Risk evaluation for AFM_1_ via consumption of raw cow milk.

Region	Age category	Av.body Wgt. (kg)	Estimated Daily Intake (EDI) (ng/ kgbw/day)	MOE	Cancer Risk (Cases/100,000 person/yr)
Greater Accra	Infants (0–11mths)	2.9	0.61	655.74	0.02
	Toddlers (12–35mths)	9.8	0.36	1111.11	0.01
	Children (36mths-10yrs)	26	0.14	2857.14	4.5 × 10^-3^
	Adolescents (11–17 yrs)	46.25	0.08	5000.00	2.58 × 10^-3^
	Adults (18–64 yrs)	60.7	0.06	6666.67	1.94 × 10^-3^
Volta	Infants (0–11mths)	2.9	1.28	312.50	0.04
	Toddler (12–35mths)	9.8	0.75	533.33	0.02
	Children (36mths-11yrs)	26	0.29	1379.31	9.37 × 10^-3^
	Adolescents (11–17 yrs)	46.25	0.16	2500.00	5.17 × 10^-3^
	Adults (18–64 yrs)	60.7	0.12	3333.33	3.88 × 10^-3^
Western	Infants (0–11mths)	2.9	2.03	197.04	0.07
	Toddlers (12–35mths)	9.8	1.20	333.33	0.04
	Children (36mths-10yrs)	26	0.45	888.89	0.014
	Adolescents (11–17 yrs)	46.3	0.25	1600.00	8.08 × 10^-3^
	Adults (18–64 yrs)	60.7	0.19	2105.26	6.14 × 10^-3^
Eastern	Infants (0–11mths)	2.9	1.58	253.16	0.05
	Toddlers (12–35mths)	9.8	0.94	425.53	0.03
	Children (36mths-10yrs)	26	0.35	1142.85	0.01
	Adolescents (11–17 yrs)	46.3	0.20	2000.00	6.46 × 10^-3^
	Adults (18–64 yrs)	60.7	0.15	2666.67	4.85 × 10^-3^

Margin of Exposure-MOE

Mean of AFM_1_- GTA= 0.26 µg/kg, VR= 0.54 µg/kg

Daily intake of milk for infants was halved (0.5 ×0.0137 kg)

Average potency of aflatoxin= 0.0323

Average Body weights were obtained from the different ranges referenced by the authors 1 µg= 1000 ng

1 µg= 1000 ng

Means of AFM_1_- WR-0.86 µg/kg, ER- 0.67 µg/kg.

Lastly, for the Eastern Region (ER), the EDI values recorded for infants, toddlers, children, adolescents, and adults were 1.58, 0.94, 0.35, 0.20, and 0.15 ng/kg bw/day respectively. MOE values recorded were 253.16, 425.53, 1142.85, 2000.00, and 2666.67, respectively. The average potency was the same as other regions, while the cancer risks were 0.05, 0.03, 0.01, 6.46 × 10^-3^ and 4.85 × 10^-3^ cases/100,000 person/yr respectively (Table 5).

## 9 Discussion

### 9.1 Occurrence of AFM_1_

Milk is consumed by most Ghanaian across all age categories; infants, toddlers, children, adolescents, and adults. Milk consumed by children and adults which are typically added to boiled tea and porridge while infants and toddlers are fed with raw milk. Considering the present findings, we detected comparatively moderate levels of AFM_1_ contamination in raw cow milk samples obtained from the regions of Southern Ghana as investigated. Our results of 0–3.52 µg/kg (3520 ng/kg) compared favorably well with some published findings of some researchers around the globe. In Ghana, Addo-Boadu [6] recorded AFM_1_ levels of range of 0.35–3.76 µg/L (350–3760 ng/L) in raw milk and milk products samples from Greater Accra Region. In Tanzania, studies conducted on raw cow milk samples revealed 83.3% (31/37) of aflatoxin contamination in cows that fed on sunflower cake in the range of 0.026–2.007 µg/kg (26–2007 ng/kg) (exceeding both Tanzania‘s and EC allowable value which is 0.05 µg/kg [69].

Several studies have reported the occurrence of AFM_1_ in milk and dairy products. Milk samples from urban centers in Kenya contained AFM_1_ up to 6800 ng/L [52]. In Sudan, 95% of milk was contaminated with AFM_1_ ranging between 220 and 6800 ng/L [28], whereas 6–527 ng/L of AFM_1_ was detected in 15% of cow milk samples from Cameroon [102]. The concentration of AFM_1_ varied between 150 and 170 ng/L in commercial and rural milk in South Africa [70], while 100% of milk samples in Nigeria contained AFM_1_ and the levels were within the range of 0.004–0.845 µg/L (4–8450 ng/l). Goncalves et al., [40] reported AFM_1_ levels in fresh bovine milk to be in the range of 0.09–3.385 µg/L (90–3385 ng/L) per their work. The least amount of data is available from African countries, nonetheless the available data suggest the highest prevalence and frequent detection levels [34]. Lower values were recorded by [110] in milk from Sudan and found 33% of the milk samples with the highest occurrence (82.4%) in cow milk (35.3% ranged between 0.05 and 0.1 μ/kg and 47.1% ranged between 0.1 and 0.15 μ/kg) and milk samples from camel in semi-intensive systems (15.6% ranged between 0.05 and 0.1 μg/kg). Again, all samples of milk from traditional nomadic systems indicated an absence of AFM_1_.

Makun et al. [67] also reported contamination of raw cow milk with AFM_1_ at levels higher than the EU permitted levels in Nigeria. In their study, contamination of raw cow milk (from nomadic cows) with AFM_1_ ranged from 0.0109 to 1.3543 µg/L (10.9–1354.3 ng/L) with and an average concentration of 0.5308 ± 0.0938 µg/L (530.8 ng/L). For commercial cow’s milk, AFM_1_ contamination ranged between 0.0464 and 0.0992 (46.4–99.2 ng/L) and 0.0584 ± 0.0052 µg/L (58 ng/L) as the mean. A similar trend regarding levels of AFM_1_ contamination of 0.05 µg/kg has also been reported in a study conducted in Brazil by Jager et al. [51]. In Egypt, Amer and Ibrahim [13] reported a prevalence of 38 positive samples with a range of 0.023–0.073 µg/L (23–73 ng/L) in raw milk. Moreover, Rahimi et al. [87] reported mean values of 0.0601, 0.0319, 0.0190, 0.0281, and 0.0301 µg/L (60.1, 31.9, 19.0, 28.1 and 30.1 ng/L) respectively for raw cow, water buffalo, camel, sheep and goat milk from Iran.

Worthy of note, milk in Europe is time and again analyzed for AFM_1_ and is also averred to be of low AFM_1_ content and perceived to be the safest [12]. In Portugal, Duarte et al. [23] reported values of n.d-0.069 µg/L (nd-69.0 ng/L) in 99.4% positive raw milk samples. In Spain, Rodríguez-Blanco et al., [89] and Cano-Sancho et al. [20] reported ranges of n.d-0.2 µg/L (n.d-200 ng/L) and 0.009–1.36 µg/L (9–1360 ng/L) respectively for raw milk samples. In Serbia, Kos et al. [58] and Tomašević et al. [103] reported ranges of 0.01–1.2 µg/L (10–1200 ng/L) and 0.09–0.145 µg/L (90–1450 ng/L) respectively for milk. Furthermore, in Croatian milk, values of 0.006—0.027 µg/L (6–27 ng/L) were reported by Bilandžić et al. [18]. AFM_1_ levels recorded in Italy by Bellio et al. [17] and De Roma et al. [21], all pointed at results below 0.05 µg/kg.

In other parts of the world, greater quantities of AFM_1_ have been reported across the globe. Lee et al. [63] from South Korea reported values of 0.22–6.9 µg/l (220–6900 ng/L) in raw milk. In Pakistan, Iqbal and Asi [50] reported values of 0.02–3.09 µg/l (20–3090 ng/L). Iha et al. [49] from Brazil, reported 83% of the milk samples tested positive for AFM_1_, in a range of 0.008–0.760 ng/g and in India, almost half of the analyzed milk was contaminated, with 44% being above EU limit [72]. Recently, Kaur et al. [53] reported AFM_1_ values of 0.314 ± 0.35 ppb in milk samples in India.

The contamination prevalence and levels of AFM_1_ in raw milk obtained in this study may be due to the reason that dairy animals kept in local dairy farms were fed with compound rations stored under poor conditions, which can be contaminated with aflatoxins. Hot and humid climatic conditions prevailing in Ghana (Table 1) are very conducive for fungal invasion, growth, and production of mycotoxins including aflatoxins in food and feed commodities [93]. Unseasonal rains and related flash floods are widespread, and this increases the moisture content of the grains and other feedstuff, and therefore its vulnerability to fungal attack. Indeed, a number of previous reports indicated the presence of high levels of aflatoxins in dairy animals, feed and ingredients from Ghana.

Moreover, most of the dairy farmers prefer to feed cereals (maize, wheat etc) or agricultural or oilseed byproducts (peanuts, soybean, etc.) to their dairy animals and such aflatoxin susceptible feed materials constitute more than 70% of cattle feed [93]. Therefore, if such high aflatoxin contaminated the feedstuff included in the diet of dairy animals, there is always a great possibility of AFM_1_ appearance in milk at high levels. Other probable factors which may play an important role in the high levels of AFM_1_ in milk in this study include poor farm management practices specially feed storage practices, no legal limits of aflatoxins exist for livestock feed and lack of knowledge among dairy farmers in relation to aflatoxins.

Aflatoxin exposure early in life has been associated with impaired growth, particularly stunting [43]. Furthermore, early exposure to aflatoxins is a potential risk for synergistic interactions with other toxins as subjects grow [14], [55]. A recent scoping review by Soriano et al., [99] has shown the presence of these aflatoxins appeared in greater proportion in kwashiorkor in children and in different organs and in biological samples including brain [81], heart [81], kidney [8], liver [80], lung [82], serum [77], stool [22], and urine [102], [46], [77], whereas in marasmic kwashiorkor they were detected in liver [47], serum [77], and urine [22], [77]. Weaning, a transition period of a child from breast milk to other sources of food, often results in a marked decrease in nutrient intake in developing countries [61]. One possible variable contributing to poor child health in developing countries is the increased exposure to aflatoxin contaminated foods following weaning [41].

Gizachew et al. [36] and Škrbić et al. [98] emphasized several factors such as geographical region, season, type and quality of feed, feed storage conditions, and processing methods and conditions are responsible for the variability of AFM_1_ in milk and dairy products. Lack of fresh forage as feed might have led to longer storage of hay or feed leading to contamination of *Aspergillus s*p. leading to AFB_1_ contamination and ultimately biotransformed into AFM_1_ [68].

### 9.2 Risk assessment

Aflatoxins are unaffected by many food processing techniques such as boiling or pasteurization, etc. as they are heat stable [59]. There is always a risk involved with their association with food or feed. Risk estimations as explained by Liu and Wu [65] as well as Kuiper-Goodman [60] are modeled to predict the magnitude of adverse health implications of mycotoxin exposure and guide food regulators to set thresholds for these toxins in foodstuffs. MOE results obtained in this study implied a high risk for infants, children, and adolescents (total aflatoxins). Our results showed a high risk for cancer due to AFM_1_ exposure from milk consumption for infants, toddlers, children, adolescents, and adults. Considering the EDI values obtained in a study by Addo-Boadu [6] in Ghana for infants, i.e., 3.679 ± 2.213 and 2.445 ± 2.001 ng/kg bw/day, it exceeded 1 ng/kg bw/day by far and indicated a serious risk to AFM_1_ through raw cow milk consumption for this age category.

Our results again agreed with the published findings of Kaur et al. [53] as they reported EDI and HCC values of 2.30 and 0.0020–0.0106, respectively. Their health risk assessment indicated that consumers, especially children, in the study area are at comparatively higher health risk to AFM_1_ owing to their low body weight and higher milk intake.

The incidence of liver cancer in Iran was 3.53 cancers per year per 10^5^ persons or 3530 cancers/yr/10^8^ persons [33] and AFM_1_ intake through yoghurt contributed 0.023–0.048 cancers/yr/10^8^ person for mean consumers and 0.028–0.069 cancers/yr/10^8^ person for high consumers. Therefore, their findings indicated AFM_1_ in yoghurt contributed a slight part of the overall incidence of liver cancer in the Iranian population. The intake of AFM_1_ and liver cancer incidence due to the consumption of this mycotoxin through yoghurt and milk have been reported in other countries including China, Spain, Greece, and Serbia [20], [45], [105]. The range of liver cancer incidence or hepatocellular carcinoma (HCC) due to AFM_1_ intake through milk and yoghurt was 0.025–0.033 case or cancers/yr/10^8^ person in China, was similar to the results of this study in Serbia and Greece was 3.6–0.4.7 and 0.7–0.9 case or cancers/yr/10^8^ person, respectively that it was higher than the current study. These distributes were related to the AFM_1_ level and consumption value of yoghurt.

Findings of studies by Serraino et al. [94] from Italy showed the EDI of AFM_1_ in different population groups was in the range of 0.025– 0.328 ng kg^-1^ body weight (bw) per day, based on the average consumption levels and weighted mean contamination of milk in the study period. The estimated fractions of HCC incidences attributable to AFM_1_ intake were 0.005 and 0.004 cases per 100,000 individuals in the 0–0.9 and 1–2.9-year age groups, respectively, and below 0.004 cases in the other age categories which posed adverse health consequences. Trevisani et al. [104] in a related study, we reported 0.011–0.057 cases/100,000 people in different age categories in an Italian population. The estimated fraction of the incidence of HCC in the Italian population projected a slight increase in cases due to milk consumption.

Contrariwise, a recent study by Njombwa et al. [73] from Malawi, reported a probable mean daily exposure to AFM_1_ for adults as 4.98 ± 7.25 ng/kg bw/day and almost double for children (8.28 ± 11.82 ng/kg bw/day). Estimated risks of AFM_1_-induced HCC associated with consumption of milk among children and adults were 0.038 and 0.023 cases per 100,000 individuals per year, respectively. Their results suggested a low risk of hepatocellular carcinoma (HCC).

In this study, EDI were moderate compared with other EDI values reported globally. This implies a reasonably significant impact of aflatoxins on the nutritional status of humans. Gong et al. [42] observed that aflatoxin exposures were to some extent linked to nutrient deficiency in humans following the suggestion that aflatoxin exposure expedites intestinal damage resulting in a decline in nutrient absorption. A noteworthy positive link between aflatoxin prevalence and zinc and vitamin A deficiency was also established by Watson et al. [109]. Furthermore, a study in Ghana reported that subjects with high exposure to the toxin were more likely to suffer deficiencies of vitamins A and E [75]. A strong association between anemia and aflatoxin has been reported in Ghana [96] showed that aflatoxin exposure may contribute partly to the high iron deficiency prevalent in children in developing countries including Ghana.

The consumption of aflatoxins at high levels in a single dose or repeatedly for a brief period induces acute intoxication, henceforward labeled aflatoxicosis in humans and animals with typical symptoms, including jaundice, lethargy, nausea, edema, hemorrhagic necrosis of liver tissues, bile duct hyperplasia, and eventually death (10–60%) subsequent to severe liver damage [84]. While there is no accord on the specific dose of aflatoxins that triggers acute toxicity in humans, it is well recognized that such a dose is highly adjustable depending on many factors, including age, gender, health and nutritional status, presence or absence of underlying factors such as chronic viral hepatitis, alcoholism, smoking, cirrhosis, exposure to hepatotoxic microcystins); and it is lowest in youngsters, as validated by the highest death rates of this age-group in aflatoxicosis outbreaks [78].

In the face of the anticipated risk of cancer incidence that can be gotten from AFM_1_ in this study, the effects of AFM_1_ on health, and especially the combined effects of mixtures of mycotoxins, the additive effects of aflatoxins, other dietary contaminants, alcohol consumption, and poor diet on cancer risk still remains largely unknown.

## 10 Conclusion

The findings of this study suggested that a moderate percentage 55% of raw cow milk samples collected in different locations of southern Ghana; Greater Accra, Volta, Western and Eastern regions of Ghana proved to have AFM_1_ contents, it further showed a public health concern considering the adverse health especially hepatocellular carcinoma (HCC) outcome of the health risk assessments in all age categories since the calculated MOEs were less than 100,000. Rigorous regulation and monitoring of livestock feeds and regular inspection of milk and milk products is vital in the lessening of AFM_1_ contamination of dairy products.

## Funding

This work received no funding.

## Consent to publish

Not applicable.

## CRediT authorship contribution statement

NKK, TA, AAB, VK-B, NOB and COT performed the experiments and wrote the manuscript. NKK, VK-B, EKE were responsible for statistical analysis. NKK, VK-B. NOB and AAB helped conceive the experiments and prepared the manuscript. NKK, TA, EKE, and COT conceived the original study and VK-B, NKK, and EKE led the sampling and study in Ghana. All authors read and approved the final manuscript.

## Declaration of Competing Interest

The authors declare that they have no known competing financial interests or personal relationships that could have appeared to influence the work reported in this paper.

## Data Availability

Data sharing is not applicable to this article as no datasets were generated or analyzed during the current study.
